# The anti-obesity properties of *Anredera cordifolia* leaf extract in rats fed a high-fat diet through inhibition of adipogenesis

**DOI:** 10.5455/javar.2023.j737

**Published:** 2023-12-31

**Authors:** Rusdiana Rusdiana, Tri Widyawati, Dina Keumala Sari, Sry Suryani Widjaja, Deddi Prima Putra

**Affiliations:** 1 Department of Biochemistry, Faculty of Medicine, Universitas Sumatera Utara, Medan, Indonesia; 2 Department of Pharmacology, Faculty of Medicine, Universitas Sumatera Utara, Medan, Indonesia; 3 Department of Nutrition, Faculty of Medicine, Universitas Sumatera Utara, Medan, Indonesia; 4 Faculty of Pharmacy, Universitas Andalas, Padang, Indonesia

**Keywords:** *Anredera cordifolia*, animal model obesity, ERK levels, PI3K levels, abdominal fat

## Abstract

**Objective:**

Various disease complications are a risk of overweight or obesity, so losing weight can reduce the risk of diseases caused by obesity. Binahong leaf ethanol extract (*Anredera cordifolia*) is a weight-loss herbal preparation.

**Aim:**

This study aims to analyze whether *A. cordifolia* extract is effective in losing weight by affecting the mechanism of adipogenesis in an animal obesity model.

**Materials and Methods:**

Animals were grouped into six groups as follows: the normal diet (K1), the negative control group (K2), the positive control group with Orlistat at a dose of 20 mg/kg BW (K3), an ethanol extract of *A. cordifolia* leaves at doses of 50 mg/kg BW (P1), 100 mg/kg BW group (P2), and 150 mg/kg BW (P3). All rats were fed a diet that consisted of high fat for eight weeks, except K1. Afterward, the treatments were given based on group distribution. Then, the rats were treated based on their groups for 4 weeks, and the high-fat diet was still given during the treatment for the control groups (K2). Anthropometric examinations such as body weight, length, and the circumference of the abdomen were measured. Metabolic parameters, including blood glucose, cholesterol levels, triglyceride levels, and abdominal fat weight, were measured using molecular parameters that measured PI3K levels and Extracellular signal-regulated kinase (ERK) in abdominal fat tissue samples using the ELISA method.

**Results:**

ERK levels of abdominal fat were lowered in the treatment group using the extract of *A. cordifolia* (50 mg/kg BW (P1) and 100 mg/kg BW (P2)) compared to the control group that was given a high-fat diet without treatment. The control group, which was fed a high-fat diet without treatment, had an average ERK level of 10.17 ± 2.98 ng/ml, P1 (50 mg/kg BW). Furthermore, when ethanol extracts were used as opposed to the control group, which received a high-fat diet without treatment, there was an increase in phosphoinositide three-kinase (PI3K) levels (K2). The control group received 9.35 ± 2.87 ng/ml, the treatment group received 100 mg/kg BW (P2) 9.48 ± 1.54 ng/ml, and the treatment group received 150 mg/kg BW (P3) 7.87 ± 1.79 ng/ml. The weight of fat in the abdomen differed between the groups that received a high-fat diet without treatment (K2) and those that received a high-fat diet with treatment (P1, P2, P3; *p* < 0.05).

**Conclusion:**

*Anredera cordifolia* extract possesses anti-obesity activities by decreasing ERK and increasing PI3K levels, as well as reducing abdominal fat weight.

## Introduction

Several noncommunicable diseases were at risk of obesity-related conditions such as hypertension, cardiovascular disease, type 2 diabetes, respiratory disease, cancer, and several other diseases and disabilities [1]. About 39% of adults worldwide are overweight, while 13% suffer from obesity. This is a cause of premature death and disability, leading to a lower quality of life and various health complications, as well as a direct or indirect increase in healthcare costs [2]. Obesity is currently affecting 78.6 million Americans (33%), and it is anticipated to affect more than half of the population by 2030 [3].

Obesity was marked by a rise in the mass of fat tissue [4] as a result of two main mechanisms, namely an increase in the number of fat cells (hyperplasia), a rise in the number of adipocytes (hypertrophy) [5], or both of these processes through the development of preadipocyte cells [6]. Weight loss principles, along with decreasing food consumption and increasing energy use [7], are anticipated to lessen overweight and obesity because they also effectively prevent adipogenesis by obstructing the process of adipocyte proliferation and differentiation [8].

Several factors, including transcription factors, signal kinases, and enzymes involved in fat metabolism, are part of the adipogenesis process [9]. Substrate of Insulin Receptor 1 (IRS-1) is triggered by insulin’s interaction with the insulin receptor (INSR), enabling regulation of differentiation through two primary pathways: the Ras/pathway and the phosphoinositide 3-kinase (PI3K)/protein kinase B (AKT) pathway. Mitogen-activated protein kinase, or MAPK [10].

The pathway of Ras/MAPK is linked to proliferation, while PI3K/AKT regulates cell division and metabolic processes in adipocytes, such as the utilization of glucose and the biosynthesis of lipids [10]. The Ras/MAPK pathway is linked to proliferation, while the PI3K/AKT pathway regulates cell growth and metabolic functions in adipocytes, such as glucose utilization and lipid biosynthesis [11].

Among the weight loss drugs known to the public are orlistat, fenfluramine, coreaserin, rimonabant, cetlistat, sibutramine, and phentermine, all of which have different weight loss properties. These drugs have various adverse health effects, such as cardiometabolic disorders, anxiety, high blood pressure and pulse rate, and depressive disorders [12]. Therefore, more efforts need to be made to find natural remedies for obesity that possess fewer adverse effects and greater effectiveness [13].

Nowadays, many traditional medications are utilized for the treatment of obesity because the accessibility and detrimental consequences of these medications can be limited [14]. Previous studies have described the extraction and usage of Binahong leaves (*Anredera cordifolia*), which are widely distributed in the highlands of North Sumatra, to treat obesity [15]. In addition, a photo marker study of Binahong leaves (*A. cordifolia*) found that its leaf ethanol extract contains alkaloids, flavonoids, saponins, tannins, triterpenoids/steroids, phenols, and quercetin compounds, as found in the identification analysis by LC-MS [16].

Adipocytes are crucial cells of fat tissue. Excess lipids (triglycerides) can build up in adipose tissue, associated with enhanced adipogenesis and lipogenesis, resulting in weight gain [17]. Obesity and its accompanying disorders are linked to adipocyte differentiation and fat storage [18, 19].

The purpose of this study is to investigate the anti-obesity activity of the ethanol extract of *A. cordifolia* leaves through a mechanism that influences the adipogenic process, as determined by measuring the extracellular signal-regulated kinase (ERK) levels and PI3K using experimental obesity animal models induced by a high-fat diet with different doses of 50, 100, and 150 mg/kg BW with orlistat as a conventional control drug.

## Material and Methods

### Ethical approval

This research was approved by the Research Ethics Committee of the Universitas Sumatra Utara (No. 705/KEP/USU/2022). The research was conducted in the Pharmacology and Therapeutics Laboratory, Department of Pharmacology, Faculty of Medicine, Universitas Sumatra Utara. The process for the preparation of ethanol extract from Binahong leaves is carried out in the Phytopharmaceuticals Laboratory, Faculty of Pharmacy, Universitas Sumatera Utara.

### Plant material and extract preparation

Powder of dried plant of *A. cordifolia* leaves 70% ethanol, aqua dest, sodium-CMC, orlistat, normal chow, high-carbohydrate chow, drinking water, amyl alcohol, ether, magnesium powder, hydrochloric acid, chloroform, Dragendorff reactant, hydrochloric acid, iron (III) chloride, sodium hydroxide, quads, chloroform, ammonia solution, 25% gelatin, Steasny reagents, sodium acetate, acetic acid anhydride, and concentrated sulfuric acid.

### Collection and authentication of plants

Binahong leaves are from the highlands of Tiganderket, Kabanjahe, North Sumatra, Indonesia, and were determined (identified) in the Herbarium Bogoriense, Biology Research Centre-LIPI Bogor, including the species *A. cordifolia* (Ten.) Steenis from the tribe Basellaceae.

### Preparation of Binahong leaf ethanol extract (A. cordifolia)

Dried leaves of Binahong were processed in a blender to create a powder. Fifty grams of the powder were macerated in 250 ml (70% ethanol) for a full day, after which they were filtered to produce a filtrate and a remnant. The residue was then macerated with 150 and 100 ml of ethanol for 24 h, then the macerated filtrate was combined and transferred to a 500 ml volumetric flask, then diluted with ethanol to the limit. The filtrate is allowed to stand for 24 h and then poured off. Frits are used to dry the filtrate after it has been evaporated using a rotary evaporator (Indonesian Herbal Pharmacopoeia, 2017).

### Phytochemical screening

Phytochemical screening was performed using standard procedures based on the analysis of metabolites present in the extract, both qualitatively and quantitatively.

### Research sample

Obese white Wistar rats (*Rattus norvegicus*, sp.) that were 8 weeks old and had a body weight of 100–150 gm were used for this study. Federer’s formula (Federer 1965, in Hanafiah 2010) is used to estimate the sample size for the experimental study. Based on the formula, 24 rats in total, split into six groups, make up the sample size. To get ready for the potential of rats dying before the study’s completion, each group added two rats, for a total of 36 rats in six groups. The inclusion criteria for the study were male Wistar rats that were 8 weeks old, weighed between 100 and 150 gm, and appeared healthy and active (eating and drinking, without any wounds, physical defects, or hair loss). Exclusion criteria included rats with additional illnesses throughout the study or who died before the end of the study.

### Maintenance and development of experimental animal models of obesity

The rats were housed in wire-mesh-covered plastic cages with dimensions of 30 cm high, 15 cm wide, and 15 cm deep. Rice husks, about 0.5–1 cm in thickness, were placed on the bottom of the cage and changed every day. The room’s lighting was precisely calibrated to 12 h luminosity. A room that ranged in temperature from 22°C to 25°C was home to three rats kept in the same cage. Production of an obese animal model with a high-fat diet 7 days before the production of the experimental animal, all these rats were acclimatized and given food in the form of standard chow (commercial pellets) and unlimited water.

### Grouping of experimental animals

Rats were arranged at random and separated into six groups after 7 days of acclimation, consisting of three control groups (K1 = receiving the standard diet, K2 = being given a high-fat diet only, K3 = being treated with orlistat while following a high-fat diet) and three groups with extract administration (three different dosages: P1 = 50-, P2 = 100-, and P3 = 150 mg/kg BW). When the models’ weight rises by more than 20% from their starting weight, they are classified as obese [20–22]. In contrast to the standard diet, which consisted of a type of pelleted diet with a dry matter nutrient content of 86%, water content of 13%, crude protein of 17.09%, fat 5.01%, crude fiber 7.01%, gross energy of 4040.09 kcal/kg, phosphorus 0.69%, ash 6%, calcium 0.09%, and organic matter of 65.04%, the sample was given a high-fat diet that included lard in the abdominal region and additionally quail yolk by tube in the morning and afternoon. The standard diet was obtained from P. Charoon Pokphan, whose nutrient composition was analyzed in 2014 at the Chemistry Laboratory of the Faculty of Animal Husbandry, College of Brawijaya, Malang. After high-fat diets were administered for eight weeks and a weight gain of over 20% was noted, the rats received the recommended treatment. This treatment was administered for 4 weeks.

After a given period of treatment, all groups were killed with 75 mg/kg BW of ketamine intraperitoneally. We collected the blood directly from the heart to examine metabolic markers such as blood glucose, cholesterol, and triglyceride levels. We open the abdomen and separate all of the fat in the abdomen by placing it in a Petri dish 22 and examine ERK and PI3K levels by ELISA methods.

### ELISA examination procedure

We collected fat tissue from the abdominal cavity, fixed in after being cleaned with regular saline and 10% neutral-buffered formalin solution, and then frozen in liquid nitrogen at the bottom of a round micro-bottle vessel, and then assessed the ERK and PI3K protein molecular.

### Analytical statistics

We used the software SPSS version 20.0 to analyze our data. We assessed the normality of each collected variable by using the Shapiro-Wilk test. The distribution of the sample variables is considered normal (*p >* 0.05) and evaluated using a one-way ANOVA. Statistical significance among groups (*p <* 0.05) was compared using post hoc LSD analysis and non-normal distribution (*p <* 0.05). We evaluated using Kruskal-Wallis and continued (*p <* 0.05) using a Mann-Whitney analysis.

## Results and Discussion

### Phytochemical screening test results of Binahong extract (A. cordifolia)

The phytochemical test results of the ethanol extraction of Binahong (*A. cordifolia*) indicate the presence of flavonoids, saponins, tannins, and steroids/triterpenoids. No alkaloids were detected during the phytochemical screening. The total flavonoid content was measured at 14.94 mg QE/gm extract using a UV-Vis spectrophotometer at a wavelength of 430 nm. Testing of antioxidant qualities by the 2,2-diphenyl-1-picrylhydrazyl (DPPH) method for protection against free radicals showed an IC50 value of 150 ppm, while the content of phenolic compounds in the Binahong leaves’ ethanol extract was 27.7019 mg GAE/gm extract.

**Table 1.** Body weight (BW) of experimental animals before and after being given a high-fat diet.

Group	BW before (gm)	BW after (gm)	*p*-value
	**Mean ± SD**	**Mean ± SD**	
K1 (*n =* 6)	106.67 ± 8.04	178.33 ± 3.72	0.027*
K2 (*n =* 6)	110.83 ± 12.69	219.67 ± 21.18	0.043*
K3 (*n =* 6)	118.67 ± 16.90	223.67 ± 17.95	0.043*
P1 (*n =* 6)	112 ± 11.98	215.83 ± 18.87	0.043*
P2 (*n =* 6)	114.67 ± 18.48	218.33 ± 22.39	0.028*
P3 (*n =* 6)	110.67 ± 15.54	208.17 ± 17.29	0.028*

*Significant (*p <* 0.05) with dependent test.

**Table 2.** Body weight (BW) before starting a high-fat diet.

Group	BW before (gm)	*p*-value
	**Mean ± SD**	
K1 (*n =* 6)	106.67 ± 8.04	
K2 (*n =* 6)	110.83 ± 12.69	0.787
K3 (*n =* 6)	118.67 ± 16.90	
P1 (*n =* 6)	112 ± 11.98	
P2 (*n =* 6)	114.67 ± 18.48	
P3 (*n =* 6)	110.67 ± 15.54	

*p >* 0.05 with one way ANOVA test.

### Anti-obesity impact of Binahong extract (A. cordifolia)

Body weight of male Wistar rats induced to become obese by a high-fat diet for 8 weeks, there was a significant increase in body weight of male white Wistar rats in each group, as determined by the dependent *t*-test (*p <* 0.05). Table 1 shows that before receiving the high-fat diets for eight weeks, male white Wistar had similar body weights, as confirmed by a one-way ANOVA test (*p* > 0.05). Table 2 is also included for reference purposes.

We found significant differences in abdominal circumference in every group both before and following administration of the high-fat diets (*p <* 0.005), as tested by the Wilcoxon test. Before receiving the high-fat diets for eight weeks, the abdominal circumference of male white Wistar rats was relatively the same, as analyzed by Kruskal-Wallis analysis (*p* > 0.05). We can see Table 3.

The body weight in animal model obesity groups after four weeks of therapy with the extract of ethanol from *A. cordifolia* showed a reduction in body weight in all groups after obtaining all the doses (50, 100, and 150 mg/kg BW). However, a reduction in body weight was not significant (*p >* 0.05). This study also showed differences in body weight in the control group that received the standard diets (K1) and the high-fat diets without treatment (K2), *p* < 0.05. The findings are displayed in Table 4.

The circumference of the abdomen (AC) in the group of rats treated with the extract Binahong at a dose of 50 or 100 mg/kg BW decreased significantly (*p <* 0.05) while using a dose of 150 mg/kg BW. The group administered Binahong extract at doses of 50 mg/kg BW and 100 mg/kg BW, which showed a significant decrease in AC (*p <* 0.05). However, the dose of 150 mg/kg BW did not show a statistically significant decrease (*p >* 0.05). On the other hand, both the normal diet group (K1) and the untreated high-fat diet group (K2) showed a significant increase in AC (K2) (*p <* 0.005). The following Table 5 displays these data.

**Table 3.** Abdominal circumference (AC) before and after being induced with a high-fat diet as a model of obesity.

Group	AC before (cm)	AC after (cm)	*p*-value	AC before (cm)	*p*-value
	**Median (min–max)**	**Median (min–max)**		**Median (min–max)**	
K1 (*n =* 6)	9.9 (9.5**–**10.1)	12.8(12**–**12.8)	0.027*	9.9 (9.5**–**10.1)	
K2 (*n =* 6)	10.05 (9.5**–**10.3)	14 (13**–**14.5)	0.043*	10.05 (9.5**–**10.3)	
K3 (*n =* 6)	10.05 (9.7**–**10.2)	14 (13.5**–**14.6)	0.043*	10.05 (9.7**–**10.2)	0.748**
P1 (*n =* 6)	10 (9.9**–**10.2)	14 (13.5**–**14.5)	0.042*	10 (9.9**–**10.2)	
P2 (*n =* 6)	10 (9.8**–**10.2)	13.7 (13.5**–**15)	0.027*	10 (9.8**–**10.2)	
P3 (*n =* 6)	9.95 (9.8**–**10.3)	13.85(12.5**–**14.3)	0.028*	9.95 (9.8**–**10.3)	

*p < 0.05 statistically significant.

*Significant (*p <* 0.05) with Wilcoxon test.

**p > 0.05 with the Kruskal-Wallis test.

**Table 4.** Body weight (BW) after being induced with a high-fat diet and being treated.

Groups	BW (obesity)	BW after treatment	*p*-value
	Median (min–max)	Median (min–max)	
K1 (*n =* 6)	178.33 ± 3.72	212 ± 2.608	0.027*
K2 (*n =* 6)	219.67 ± 21.18	252 ± 32.334	0.043*
K3 (*n =* 6)	223.67 ± 17.95	203 ± 18.641	0.068
P1 (*n =* 6)	215.83 ± 18.87	209.80 ± 13.809	0.686
P2 (*n =* 6)	218.33 ± 22.39	212.17 ± 21.274	0.075
P3 (*n =* 6)	208.17 ± 17.29	207.17 ± 12.416	0.528

*Significant (*p <* 0.05) with dependent test.

**Table 5.** The abdominal circumference (AC) after a high-fat diet induction and treatment.

Groups	AC obesity	AC after treatment	*p*-value
	Median (min–max)	Median (min–max)	
K1 (*n =* 6)	12.8(12**–**12.8)	13.6 (13.4**–**13.8)	0.026*
K2 (*n =* 6)	14 (13**–**14.5)	16.5 (15**–**17)	0.041*
K3 (*n =* 6)	14 (13.5**–**14.6)	14 (13.2**–**14)	0.063
P1 (*n =* 6)	14 (13.5**–**14.5)	13.7 (13.3**–**14.4)	0.041*
P2 (*n =* 6)	13.7 (13.5**–**15)	13.35 (13.2**–**13.5)	0.043*
P3 (*n =* 6)	13.85(12.5**–**14.3)	13.4 (13**–**14)	0.345

*p < 0.05 statistically significant.

*Significant (*p <* 0.05) with Wilcoxon test.

**Table 6.** ERK levels, PI3 K levels in adipocytes, and Post Hoc test analysis PI3K.

ERK Levels(ng/ml) adipose cells			PI3K levels (ng/ml) Adipose cells		Post Hoc PI3K				
Group	Mean ± SD	*p*-value	Mean ± SD	*p*-value	K2	K3	P1	P2	P3
K1	9.012 ± 2.38		11.70 ± 1.73		0.002**	0.357	0.127	0.131	0.012
K2	10.17 ± 2.98		6.46 ± 3.19			0.020**	0.074	0.052	0.352
K3	11.11 ± 1.40	0.349	10.30 ± 3.45	0.026*			0.547	0.928	0.330
P1	8.62 ± 1.28		9.35 ± 2.88					0.928	0.265
P2	8.97 ± 1.29		9.49 ± 1.54						0.265
P3	10.22 ± 2.30		7.87 ± 1.79						

*p < 0.05 statistically significant.

One-way ANOVA test.

Examination of ERK and PI3K levels derived from fat in the stomach using the ELISA method. The results are presented in Table 6. However, in this study, it can be seen that the ERK levels were reduced in the treatment groups (P1) and (P2) but not in (P3) compared to the untreated control group in the standard diet group (K1) and the high-fat diet group (K2). Whereas a mean PI3K level, also derived from abdominal fat, showed a difference in significant PI3K levels in the adipocytes among the groups (*p <* 0.05). A *post-hoc* LSD analysis showed a significant difference in PI3 K levels between the control group of the standard diets (K1) and the control group of the high-fat diets (K2) (*p <* 0.05) and the control group with orlistat (K3) (*p <* 0.05). There were increased PI3K levels in the groups (P1) and (P2). However, the comparison of this elevation with the control group’s high-fat diet (K2) did not reveal any significant statistical difference.

**Table 7.** Examination of BSLs and lipid profiles at the group of rat obesity model after 4 weeks of treatment.

Groups	BSL after (ng/dl) *p*-value	Trygliceride after (ng/dL)	Cholesterol (ng/dl)	*p*-value
	Mean (SD)	Mean (SD) *p*-value	Median (min-max)	
K1 (*n =* 6)	206.61 ± 98.84	112.71 ± 76.98	14.27 (10.54**–**20.54)	
K2 (*n =* 6)	225.51 ± 60.93	126.73 ± 27.03	30.68 (12.36**–**93.31)	
K3 (*n =* 6)	186.74 ± 31.62 0.528	121.22 ± 56.69 0.461	13.25(10**–**16.78)	0.396
P1 (*n =* 6)	207.29 ± 47.16	84.10 ± 56.69	12.80 (10.89**–**20)	
P2 (*n =* 6)	200.73 ± 51.95	82.51 ± 35.16	10.99 (8.98**–**17.96)	
P3 (*n =* 6)	257.37 ± 62.94	88.20 ± 34.66	14.43 (10.83**–**16.37)	

With one-way ANOVA test *p <* 0.005 statistically significant.

This study showed that the decreased blood sugar level (BSL) in the treatment groups (P1) and (P2) but statistically showed no significant decrease (*p >* 0.05), reduced triglyceride and cholesterol levels at all groups (P1, P2, P3) but not a significant decrease. The research results are presented in Table 7.

The purpose of this study is to see how an extract of ethanol from *Anredera* affects white Wistar’s high-fat diet. In the rat group, high-fat diet (HFD) administration caused an increase in body weight of more than 20% of body weight before being given a diet for eight weeks.

The study showed a difference in body weight in the control group (K1) and (K2) and also in all treatment groups (*p* < 0.05). This showed that weight gain with a high-fat diet resulted in a higher body weight compared with the standard diet. This study is based on previous studies [23]. A high-fat diet is widely used to promote obesity in experimental settings in animal models for eight weeks [24] and produces adverse effects [25], so we can find out that diet is a major factor in the obesity epidemic [26]. Consumption of foods from high-fat diets can lead to obesity development in humans because it increases body adipocytes, as evidenced by the weight of abdominal fat in the standard diet group, which is smaller than the high-fat diet group, and promotes the onset of glucose intolerance and hypertension [27].

This study discovered that using ethanol from *Anredera,* an anti-obesity substance, reduced body weight at all doses (50, 100, and 150 mg/kg BW). For four weeks, a dose of 50 mg decreased body weight by 6.03 gm, a dose of 100 mg/kg reduced body weight by 6.16 gm, and a dose of 150 mg/kg decreased body weight by 1 gm. In this study, using a dose of 100 mg/kg BW, BW lost weight more than the other dosage for four weeks, the same as in previous studies [28].

This investigation also discovered that the extract of ethanol from *A. cordifolia* at 50 and 100 mg/kg BW dosages caused a significant decrease in terms (*p <* 0.05), but abdominal circumference was not significantly reduced (*p >* 0.05) at a 150 mg/kg BW dosage. A dose of 100 mg/kg BW reduced abdomen circumference more than a dose of 50 mg/kg BW. This research showed a significant increase in abdominal circumference (*p <* 0.05) in the control groups (K1) and (K2) because these two groups were not given treatment, indicating that there was a greater rise in abdomen circumference given a high-fat diet compared to the group given the standard feed. This demonstrates that anthropometric abdominal circumference measurements have a strong correlation with the fat compartment [29]. Therefore, when compared to orlistat, using Binahong (*A. cordifolia*) extract can reduce fat in the abdomen as well as body weight. This was also indicated by the distinction in the weight of the abdominal fat found in the control group on a high-fat diet without treatment in comparison to treatment at all doses (*p <* 0.05). We know that central obesity is a fat accumulation that can be harmful to health, and it can be caused by excess subcutaneous and visceral fat as a result of an energy inconsistency between nutritional consumption and a lack of physical activity [30].

In previous studies, increased ERK activity resulted in hypertrophy of adipose tissue in rats given high-fat diets. The research found that the control group with high-fat diets (K2) found greater body weight in comparison to the standard diet control group (K1), while in the treatment group, there was a decrease in ERK levels (P1, P2), compared with the ERK levels of the high-fat diet control group that did not receive treatment (K2). A previous study showed that the ERK signal is activated throughout the initial process of fat formation (adipogenesis), increasing ERK levels. Previous studies have suggested that ERK activation plays a role in fat formation (adipogenesis) [31]. This occurrence can be explained by the fact that ERK signals are required in the beginning stages of adipocyte cell differentiation if the absence of ERK causes resistance to adipocyte development or disruption of the process of adiposity development under the administration of pre-adipocyte HFD from the mouse group and embryonic fibroblasts showing disruption of the adipogenesis process [32].

Even so, in accordance with *in vitro* research with contradicting findings, it was found that sustained adipogenesis would decrease if ERK was activated because peroxisome proliferator-activated receptor (PPAR)γ expression can be inhibited by prolonged stimulation of the sustained activation through MAPK40-mediated phosphorylation [33].

The insulin signaling pathway depends on the PI3K/AKT pathway. A disruption in regulating this signaling correlates with the occurrence of insulin resistance and obesity [34]. There is an adverse relationship between PI3K and AKT. It has been discovered that exercise level and percent of body fat are related in either humans or model animals, and AKT may contribute to insulin resistance in the obese population [35,36]. In this study, the group of patients treated with doses of 50 and 100 mg/kg BW of extract ethanol Binahong increased levels of PI3K, increased the uptake of glucose in adipose tissue, and decreased BSLs in the plasm.

Finally, we can provide an anti-obesity theoretical foundation effect of *Anredera* extract via suppressing the adipogenesis process with evidence of decreased ERK levels and then increased levels of PI3K to decrease the BSL.

## Conclusion

Ethanol extract from Binahong (*Anredera*) can reduce body weight, reduce abdominal circumference, and then reduce the BSL for 4 weeks of administration. Weight loss is related to a decrease in the adipogenesis process through a decrease in ERK levels in adipose tissue and a decrease in BSLs due to an increase in PI3K levels, thereby increasing glucose uptake in adipose tissue.

## List of abbreviations

SPSS, Statistical package for the Social Sciences; Anova, analysis of variance; LSD, least significant difference IRS-1, insulin receptor substrate 1; PI3K/AKT, phosphoinositide kinase; ERK, extracellular signal-regulated kinase; BSL, Blod Sugar Level; HFD, high-fat diet; PPARγ, peroxisome proliferator-activated receptor; MAPK, mitogen-activated protein kinase.
